# Geographic Information Systems (GIS) in Assessing Dental Health

**DOI:** 10.3390/ijerph7052423

**Published:** 2010-05-25

**Authors:** Stela M. Pereira, Gláucia M.B. Ambrosano, Karine L. Cortellazzi, Elaine P.S. Tagliaferro, Carlos A. Vettorazzi, Sílvio F.B. Ferraz, Marcelo C. Meneghim, Antonio C. Pereira

**Affiliations:** 1 Department of Community Dentistry, Piracicaba Dental School, P.O. BOX 52. University of Campinas—UNICAMP, Piracicaba, SP 13414-903, Brazil; E-Mails: glaucia@fop.unicamp.br (G.M.B.A.); karinecortellazzi@gmail.com (K.L.C.); epstag@gmail.com (E.P.S.T.); meneghim@fop.unicamp.br (M.C.M.); apereira@fop.unicamp.br (A.C.P.); 2 Department of Public Health, Lavras University Center—Unilavras, Lavras, MG 37200-000, Brazil; 3 Department of Rural Engineering - ESALQ, University of Sao Paulo, Piracicaba, São Paulo, SP 13418-900, Brazil; E-Mails: cavettor@esalq.usp.br (C.A.V.); sfbferra@esalq.usp.br (S.F.B.F.)

**Keywords:** spatial analyses, geographic information systems, dental caries

## Abstract

The present study investigated the distribution profile of dental caries and its association with areas of social deprivation at the individual and contextual level. The cluster sample consisted of 1,002 12-year-old schoolchildren from Piracicaba, SP, Brazil. The DMFT Index was used for dental caries and the Care Index was used to determine access to dental services. On the individual level, variables were associated with a better oral status. On the contextual level, areas were not associated with oral status. However, maps enabled determining that the central districts have better social and oral conditions than the deprived outlying districts.

## 1. Introduction

For a better understanding of the oral health/disease process, it becomes interesting to explore the relationships between space and Community Health. Today, geographic space is understood as an active environment, a receptor of social processes and an activator of these processes [1]. Detailed analysis of the pattern of inequality and spatial distribution of oral diseases is fundamental for the allocation of resources to areas with the greatest social privation, leading to greater efforts to address the problems [2].

Within this new approach, geoprocessing tools have appeared, and among them Geographic Information Systems (GIS) stand out as valuable technology in the exploration of these relationships, contributing to a better understanding between the environment and health [3], at the same time in which they provide health services with rapid understanding of the locations where the problems occur with greater frequency, facilitating the continuous process of planning, monitoring and evaluating oral health services.

There has been a great deal of discussion about the most adequate strategies for evaluating and intervening in the process of inequality in the distribution of oral diseases observed all over the world [4–6], with emphasis on the populational approach, whose fundamental principle is to rescue the role of the sociocultural environment in the distribution and determination of diseases; and approach that has generated great interest among researchers [7]. Recent studies have shown the importance of the use of Geographic Information Systems (GIS) in this type of strategy, as a method increasingly accepted and used in epidemiological studies, providing important information with regards to analysis of the geographic distribution of diseases, their associations with social, economic and environmental factors, and pathogenic agents, as well as elucidating the mechanisms of diseases [8,9]. GISs are still infrequently used in public health, and even more rarely in dental studies, however, the method has attained an outstanding place among professionals in the area due to the innovative information it can offer with regards to understanding, planning, monitoring and allocation of health resources.

In countries such as Brazil, this tool facilitates the planning of services, as, in some areas, the physical environment does not present a clear division between the different social strata, therefore, it runs into situations and realities that are common in present times, in which disorderly expansion of urban areas gives space to a mixture of realities. Luxury condominiums can easily be seen among areas of social privation. In Brazil, this is an increasingly common scenario. The contextual data attributed to an elite cluster also includes individuals of an inferior socio-economic level. This situation leads public health researchers to the use of tools such as GIS that facilitate the visualization of this scenario.

According to these precepts, it becomes important for these studies to begin to use not only these high technology tools, but also to analyze the data in order to contemplate the intricate relationships between the individual and the environment, taking into account the hierarchy of complexity and multiple interactions among the different levels studied, conjecturing the impossibility of separating the individual from his territory [1].

Thus the aim of this study was to evaluate the distribution profile of dental caries and its associations with areas of social deprivation at two levels, individual and social determinants of clusters, by means of Geographic Information Systems and Multilevel Analysis.

## 2. Methods

### 2.1. Ethical Aspects

This study was approved by the Research Ethics Committee of the Piracicaba Dentistry School, State University of Campinas/UNICAMP (protocol: 098/2006).

### 2.2. Sample

To calculate the sample size the data of a previous study conducted in the municipality [10] were considered, admitting a sampling error of 7%, level of confidence of 95% and design error equal to 2. The schools were selected by means of probabilistic sampling by cluster. The sample was composed of 1,002 school children aged 12 years, enrolled at 25 public and private schools in the municipality, distributed throughout 18 districts. The city of Piracicaba has around 360,000 inhabitants.

### 2.3. Inclusion and Exclusion Criteria

Individuals with signed terms of informed consent from their parents/guardians and no systemic diseases were included (one child had a kidney problem and was in dialysis treatment). Individuals who refused to participate, those who did not have a signed term of informed consent and those with systemic diseases were excluded from the study.

### 2.4. Exams

All the exams were carried out by only one previously calibrated examiner (Kappa > 0.89). The entire examiner calibration process was conducted by a standard examiner (Gold Standard) experienced in epidemiological surveys, and the theoretical-practical activities of the training exercises consisted of a total of seven periods of four hours. Ten percent of the sample was reexamined to calculate the intra-examiner error in the second calibration period after the interval of one day. The training exercises were carried out over seven four-hour sessions. The calibration process (count and analysis of examiner’s errors without contact or conversation with the gold standard) was performed in two steps. The first step was the assessment of errors by the examiner and the gold standard (inter-examiner calibration). The second step was the assessment of errors by the same examiner, who performed a second data collection on 10% of the sample for comparison purposes (intra-examiner calibration).

The exams were conducted in accordance with the recommendations of the World Health Organization for the codes and criteria [11]. All the schoolchildren received a brush kit, containing toothbrush, toothpaste and dental floss, and were instructed during tooth brushing by a Dental Hygienist. The exams were performed in outdoor setting, with the individuals seated on chairs, under natural light, use of a ball point probe, oral mirror, and with the aid of air-drying using a portable mini-compressor. Due to the distances between neighborhoods, 40 students were examined per day on average.

### 2.5. Indexes and Variables Used

The DMFT Index (number of decayed, missing and filled teeth) was used for dental caries, and the Care Index, to measure access to dental services, by the equation: (FT/DMFT) × 100 [12]. The following were considered as independent variables at the first individual level: monthly family income, people living in the household, home ownership, car ownership, father’s and mother’s educational levels, oral hygiene habits and visits to the dentist, obtained by means of a previously validated semi-structured questionnaire [10]. The variables percentage of heads of families without income, and percentage of illiterate heads of families, were used as variables for the clusters.

### 2.6. Data Analysis

Univariate analysis was used to verify the influence of the socio-economic and behavioral variables on oral health using the Chi-square test (χ^2^) at 5% level of significance. The goal of multilevel regression analysis was to identify variables that would be associated with areas of social privation at two levels, individual and contextual (Districts). In this analysis, the “DMFT Index” and “Care Index” were considered as response-variables; as demographic variables of the cluster “the percentage of heads of families without income” and “the percentage of illiterate heads of families” were considered, and as variable of individuals, the socio-economic and behavioral information was used. The purpose of the multilevel analysis was to minimize the discrepancies between the variables of the individuals (at the first level) using the residue of this first level to evaluate contextual variables (of the districts). By means of the multilevel regression model, the odds ratios and their respective intervals of confidence of 95% were estimated. The DMFT Index (dependent variable) was dichotomized according to the median (Med = 0) and the Care Index was dichotomized according to the tercile. In addition, a Spearman Correlation was performed between income (level of subjects) and the variables of the clusters. All statistical tests were performed using the SAS software (SAS Institute Inc. 9.1, 2003) at 5% significance level. For spatial analysis the Geographic Information Systems were set up, and the thematic maps being constructed with the aid of ArcView 3.1 Software.

## 3. Results

The results of the present study, by means of analysis of the DMFT Index, demonstrated that the lower the illiteracy indexes, the lower the rates of “percentage heads of families receiving no income” the less was the severity of caries disease (Table 1).

In Table 2, gender, income, people living in the household, father’s and mother’s educational level, visits to the dentist and car ownership variables presented association with the DMFT Index (p < 0.15) and were tested in the multilevel model. As regards the analysis of the dependant variable Care Index (Table 2), the following variables were significantly associated: income, people living in the household, father’s and mother’s educational level, visits to the dentist, home ownership, tooth brushing frequency, car ownership and onset of tooth brushing (p < 0.15). These variables were tested in the respective multilevel analyses.

In the Multilevel Regression Analysis, at “individual level”, students with lower income (OR = 1.8; CI = 1.0–3.6), more people living in the household (OR = 1.4; CI = 1.0–1.8), lower number of visits to the dentist (OR = 1.8; CI = 1.3–2.4), father’s (OR = 1.7; CI = 1.0–3.3) and mother’s (OR = 1.7; CI = 1.1–1.3) lower educational level were more likely to present a higher DMFT (Table 3). The individuals with higher income (OR = 3.9; CI = 0.8–17.9) and more visits to the dentist (OR = 4.7; CI = 2.9–7.7) showed the best Care Index (table 4). At a conglomerate level, areas with social deprivation were not associated with the DMFT and the Care Indexes (Tables 3 and 4). In addition, a Spearman Correlation was performed between income (individual level) and the variables that characterized the clusters. Correlation was observed between the percentage of heads of families receiving no income and percentage of heads of families that were illiterate (p < 0.0001), whereas with regard to income (variable of subjects) this correlation could not be observed (p = 1.0000).

**Figure 1.**
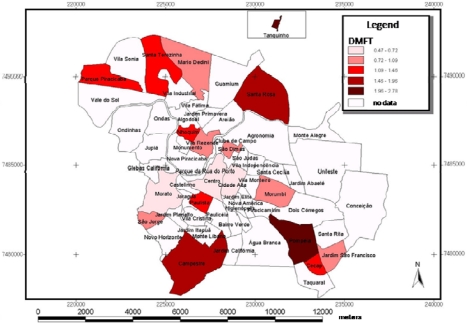
Distribution of the DMFT Index in the sampled districts.

**Figure 2.**
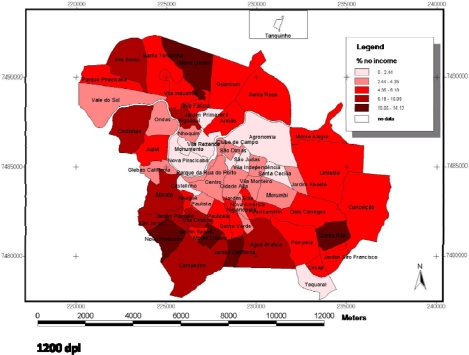
Smaller map containing the demographic data of percentage of heads of families receiving no income in the districts (IPPLAP—Research and Planning Institute of Piracicaba).

**Figure 3.**
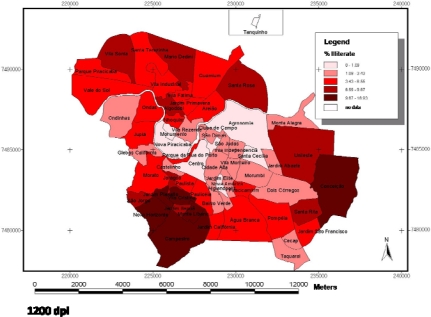
Smaller map containing percentage of heads of families that was illiterate in the districts (IPPLAP – Research and Planning Institute of Piracicaba).

**Figure 4.**
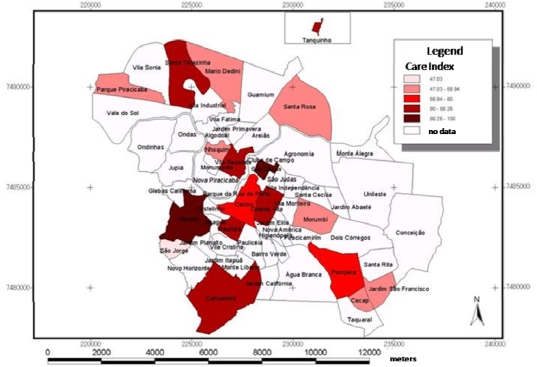
Distribution of the Care Index in the sampled districts.

In Figure 1, which presents the maps related to the DMFT Index (larger map), and in relation to the variables percentage of heads of families receiving no income (Figure 2) and percentage of heads of families that were illiterate (Figure 3), presented in the smaller maps, it is possible to observe that the central districts have better social and oral conditions, however, it is important to point out that this difference was not significant in the Multilevel analysis. In a similar manner, Figure 4 presents the maps related to the Care Index (larger map), and it is possible to note a similar trend, in which the best conditions are visualized in the central areas, however, this condition was also not significant. The smaller maps containing the information related to the social context (variables of the clusters, Figures 2 and 3) were included with the purpose of facilitating understanding and comparison of the two levels studied (contextual and individual).

## 4. Discussion

Initially, it is important to define the basic terminology more commonly used, in order to understand it better. Data geoprocessing involves the entire process, starting with data collection through to making the maps. Whereas, a Geographic Information System can be defined as a computational system provided with four groups of aptitudes to work with georeferenced data input, management, manipulation and analysis, and output [13].

The use of spatial methodology for health areas has been observed, especially in the medical area [14–17]. In dentistry, studies are recent and have demonstrated important results for public health planning [2,18]. Furthermore, geographic indicators are capable of discriminating small areas of social privation [19], in which the reduction of caries prevalence was lower.

The distribution of dental caries in the municipality, by means of maps (Figure 1), follows the same distribution trend as the social and economic variables previously observed (Figures 2 and 3). The more central districts have better oral health conditions, which can be observed both for the DMFT Index (Figure 1), and the Care Index (Figure 4). Although this information is capable of being observed in the maps, the multilevel analysis did not show that there was any significant association between oral conditions and the characteristics of the clusters in the present study.

In the dentistry literature studies that use spatial analysis are scarce, nevertheless, the findings of the present study corroborate the study of Antunes [6] conducted in the city of São Paulo, which found high levels of dental caries in areas of social privation (outlying deprived areas), while the individuals that were at lower risk occupied the central portion of the municipality, and the same occurred as regards the treatment requirements, in which the greatest necessities were verified in the (outlying) deprived areas. Nevertheless, it is important to point out that the study Antunes [6] was developed in São Paulo, which is the largest city in Brazil, concentrating around 11 million inhabitants, while Piracicaba has around 360,000 residents [20]. The differences as regards the number of inhabitants and extent of the municipalities are discrepant, however, the non-uniform distribution profile of the disease observed all over the world [21] occurs in a similar manner. These findings support the discussions related to heterogeneity of the oral manifestations and support the planning of actions in other municipalities.

Another important aspect is the role of Multilevel Regression analysis in recent studies [22,23]. This analysis provides a more faithful analysis of the relationships between the environment and individuals, not separating them completely as the commoner analyses normally do. The analysis referred to takes into consideration the two levels considered: individuals and contextual data (cluster), combining them with the purpose of minimizing the discrepancies among the variables that were collected from the individuals (at a first level) and the variables of the entire context in which these groups are included.

Concomitantly with the reduction in the prevalence of dental caries, the growing social inequality of the distribution of the disease has become clearer [24]. This process is known as “polarization” of the disease, in which small portions of the population concentrate the greater part of the disease, which is normally recognized as socially deprived population groups.

Studies directed towards delimitation of the areas of greater risk for diseases and treatment needs involving, in addition to clinical variables, socio-economic, behavioral and geographic characteristics, would be extremely important to public health services, in order to optimize the allocation of financial and human resources. In addition to the importance of mapping to analyze the distribution of diseases and their possible associations with environmental variables, the risk map would be a tool enabling easy identification and immediate understanding of the geographical areas where there is greater and more severe occurrence of health problems, and it would also be valuable to the public health services for obtaining subsidies to organize health programs.

In this sense, in “spatialization” of the oral health-disease process, the Public Health Services could find a relationship between occurrences and determinants, and adopt epidemiology as a privileged referential in the study of this relationship [1].

The limitation of the present study is that in some areas, the physical environment of the municipality does not present a clear division between the different social strata, therefore, it runs into situations and realities that are common in present times, in which disorderly expansion of urban areas gives space to a mixture of realities, as luxury condominiums coexist with areas of social privation. In Brazil, this is an increasingly common scenario. As regards the municipality of Piracicaba, this panorama is still evolving, however, it can already be seen as a limitation of this study, since some of the elite areas studied were mixed in with outlying deprived areas of the municipality, there being no distinction between their boundaries. In other words, the contextual data attributed to an elite cluster by the municipal authorities [25], also includes individuals of an inferior socio-economic level. The non-association between socially private areas and the oral health conditions can be a reflection of this situation.

The findings demonstrated a univariate association between socio-economic variables and the educational level of the parents and oral health, but did not demonstrate association between the private areas and the oral conditions in the multilevel model. In a similar manner, a study conducted in Scottish adults [22] did not find a significant association between the deprivation area and the oral health of adults, suggesting further multilevel research exploring the relationship between deprivation area and oral health using a much higher number of participants and geographic areas in a prospective longitudinal design.

These data reinforce the hypothesis that the variables of the districts are not significantly associated to the oral health conditions in the municipality, in spite of visualization of the maps showing differences between areas with social privation and those without social privation. On the other hand, the variable income (subject level) was shown to be a strong indicator of risk for dental caries, which evidences the previous discussion, demonstrating that today, various realities occupy the same space, and consequently, families from different income levels live in the same districts. By means of the present study, it is possible to conclude that at individual level, social and economic variables were associated with a higher prevalence of the disease; however, this relationship was not observed at territorial level.

## Figures and Tables

**Table 1.** Frequency of the DMFT Index and Care Index (mean, standard deviation and median) as a function of the type of school and districts.

				**DMFT**		**Care Index**	
				
**School**	**District**	**% No income**	**% Illiterate**	**Mean (SD)**	**Median**	**Mean (SD)**	**Median**
Private	V.Rezende	2.15	2.06	0.12 (0.33)	0.00	100.00 (0.00)	100.00
	Alto	2.95	1.64	0.48 (0.75)	0.00	85.71 (37.80)	100.00
	S. Dimas	2.77	2.21	0.94 (1.32)	0.00	100.00 (0.00)	100.00
	Morato	7.54	5.95	0.47 (1.02)	0.00	100.00 (0.00)	100.00
	Centro	3.29	0.75	0.55 (0.69)	0.00	80.00 (44.72)	100.00
Public	S.Francisco	5.73	5.63	1.09 (1.59)	0.00	67.65 (40.06)	90.0
	S.Rosa	5.81	7.69	1.67 (1.96)	1.00	67.94 (38.98)	80.0
	V.Rezende	2.15	2.06	1.17 (2.06)	0.00	79.29 (35.38)	100.0
	Alto	2.95	1.64	1.28 (1.68)	0.00	75.36 (37.35)	100.0
	CECAP	5.42	3.05	1.19 (1.62	1.00	68.94 (42.50)	100.0
	Nho Quim	4.08	4.06	1.43 (1.60)	1.00	68.44 (44.36)	100.0
	Morumbi	4.24	2.92	1.09 (1.51)	0.00	64.44 (43.01)	75.0
	S.Jorge	7.76	9.66	1.09 (1.89)	0.00	47.03 (47.31)	45.0
	S.Dimas	2.77	2.21	0.91 (1.47)	0.00	88.63 (26.53)	100.0
	B.Lenheiro	13.55	7.77	1.00 (1.50)	0.00	59.71 (46.79)	80.0
	Tanquinho	-	-	2.33 (1.69)	2.00	88.26 (29.95)	100.0
	Campestre	8.47	11.62	1.96 (1.70)	2.00	86.38 (32.26)	100.0
	S.Terezinha	6.18	6.55	1.29 (1.53)	1.00	84.14 (28.82)	100.00
	Paulista	3.14	4.72	1.46 (1.76)	1.00	82.73 (36.68)	100.0
	Pompéia	5.72	4.8	2.78 (3.32)	2.00	77.97 (37.40)	100.0
	P.Piracicaba	6.04	5.42	1.26 (1.91)	0.00	65.29 (44.15)	100.0

**Table 2.** Bivariate association between DMFT (dichotomization by the median) and gender, socio-economic characteristics and behavioral variables related to DMFT and Care Index at the first level (subjects).

**Variable**	**DMFT = 0 n (%)**	**DMFT > 0 n(%)**	**p-value**	**CI# ≤ 75% n (%)**	**CI# > 75% n (%)**	**p-value**
**First level: subjects**						
Gender						
Female	289(50.09%)	288(49.91%)	0.0216	88(30.56)	200(69.44)	0.6427
Male	244(57.41)	181(42.59%)		59(32.60)	122(67.40)	
Monthly family icome up to 3 minimum wages*	463(51.16%)	442(48.84%)	0.0002	144(32.58)	298(67.42)	0.0495
> 3 minimum wages	60(72.29%)	23(27.71%)		3 (13.04)	20(86.96)	
People living in the household						
≤4 people	291(55.85%)	230(44.15%)	0.0700	61(26.52)	169(73.48)	0.0195
>4 people	236(50.11%)	235(49.89%)		86(36.60)	149(63.40)	
Father’s education						
Complete middle-school	200(45.05%)	244 (54.95%)	<0.0001	84(34.43)	160(65.57)	0.0503
Complete high school	143(59.58%)	97(40.42%)		23(23.71)	74(76.29)	
Complete undergraduate	89(68.99%)	40(31.01%)		8(20.00)	32(80.00)	
Mother’s education						
Complete middle-school	283(47.56%)	312(52.44%)	<0.0001	108 (34.62)	204(65.38)	0.0172
Complete high school	175(61.40%)	110 (38.60%)		31 (28.18)	79(71.82)	
Complete undergraduate	69(61.61%)	43(38.39%)		6(4.14)	37(86.08)	
Visits to the dentist						
Never/Irregularly	246(57.75%)	180 (42.25%)	0.0060	91(50.56)	89(49.44)	<0.0001
Regularly	274(48.93%)	286 (51.07%)		55(19.23)	231(80.77)	
Home ownership						
Yes	345(54.59%)	287(45.41%)	0.2341	83(28.92)	204(71.08)	0.1329
No	185(50.68%)	180(49.32%)		64(35.56)	116(64.44)	
Car ownership						
No car	202(47.64%)	222(52.36%)	0.0034	81(36.49)	141(63.51)	0.0214
≥1	320(57.04%)	241(42.96%)		64(26.56)	177(73.44)	
Toothbrushing frequency						
≤ once/day	65(51.59%)	61(48.41%)	0.7136	26(42.62)	355(57.38)	0.0401
> twice/day	464(53.33%)	406(46.67%)		120(29.56)	286(70.44)	
Onset of toothbrushing						
≤1 year old	432(53.93%)	369(46.07%)	0.2966	107(29.00)	262(71.00)	0.0174
>1 year old	95(49.74%)	96(50.26%)		40(27.21)	56(58.33)	

* Minimum wage at the time of the data collection, approximately US$163.55

# Care Index.

**Table 3.** Multilevel logistic regression model with DMFT as dependent variable.

	**Estimate**	**SE**	**Adjusted OR**	**95%CI**	**p**
**FIRST LEVEL: INDIVIDUAL CHARACTERISTICS**					
Monthly family icome:					
up to 3 minimum wages	0.3229	0.1602	1.8	1.0−3.6	0.0313
> 3 minimum wages	Reference				
People living in the household:					
> 4 people	0.1547	0.0756	1.4	1.0−1.8	0.0344
≤ 4 people	Reference				
Father’s education :					
Complete middle-school	−0.3410	0.1924	0.7	0.5−1.05	0.0763
Complete high school	−0.5466	0.2824	0.6	0.3−1.0	0.0532
Complete undergraduate	Reference				
Mother’s education:					
Complete middle-school	−0.4706	0.1947	0.6	0.4−0.9	0.0157
Complete high school	−0.1688	0.2899	0.8	0.5−1.4	0.5604
Complete undergraduate	Reference				
Visits to the dentist:					
Never/Irregularly	0.2860	0.0792	1.8	1.3−2.4	0.0003
Regularly	Reference				
-2 loglikelihood (first level)	1015.528				
	Estimate	SE	β	p	
**SECOND LEVEL: CLUSTER CHARACTERISTICS**					
% No income/cluster	0.00876	0.0516	−0.0580	0.8651	
% Illiterate/cluster	−0.0445	0.0504	0.1259	0.2027	
-2 loglikelihood (full model)	1044.459				

**Table 4.** Multilevel logistic regression model with Care Index as dependent variable.

	Estimate	SE	Adjusted OR (IC > 75%)	95%CI	p
**FIRST LEVEL: INDIVIDUAL CHARACTERISTICS**					
Monthly family icome up to 3 minimum wages	Reference				
>3 minimum wages	0.6799	0.3902	3.9	0.84−17.9	0.0601
Visits to dentist:					
Never/Irregularly	Reference				
Regularly	0.7794	0.1229	4.7	2.9−7.7	<0.0001
−2 loglikelihood (first level)	577.178				
	Estimate	SE	β	p	
**SECOND LEVEL: CLUSTER CHARACTERISTICS**					
% No income/cluster	−0.3049	0.1705	−2.0138	0.0960	
% Illiterate/cluster	0.1606	0.1705	−0.2533	0.3492	
−2 loglikelihood (full model)	554.191				
